# The Correlation of Regional Microstructure and Mechanics of the Cervical Articular Process in Adults

**DOI:** 10.3390/ma14216409

**Published:** 2021-10-26

**Authors:** Huimei Feng, Yuan Ma, Stephen Jia Wang, Shaojie Zhang, Zhijun Li

**Affiliations:** 1 Department of Anatomy, School of Basic Medical Sciences, Inner Mongolia Medical University, Hohhot 010059, China; fhm19912019@hotmail.com; 2 Inner Mongolia Medical University Graduate School, Hohhot 010059, China; 3 Digital Medical Center, Inner Mongolia Medical University, Hohhot 010059, China; my15947125196@163.com; 4 School of Design, Hong Kong Polytechnic University, Hong Kong, China; Stephen.j.wang@polyu.edu.hk

**Keywords:** cervical articular process strength, micro-CT, regional microstructure, finite element analysis, mechanical model

## Abstract

Purpose: Using micro-CT and finite element analysis to establish regional variation microarchitectures and correlation with mechanical properties of cervical articular facet trabecular bone to predict cervical spine security and material properties. Methods: A total of 144 cervical articular processes (each articular was separate to four region of interest (ROI), superior-anterior (SA), superior-posterior (SP), inferior-anterior (IA), and inferior-posterior (IP) regions) specimens with a volume of 5 × 5 × 5 mm^3^ were scanned by micro-CT, and allowable stress and other mechanical properties parameters in each region were calculated after mechanical testing, then the effectiveness was verified of finite element models by ABAQUS software. Results: Maximum and minimum values of C2–C7 articular processes and regions are C5 and C7 level, SA and SP regions for bone volume fraction (BV/TV) and trabecular thickness (Tb.Th), whose variation tendency is similar to the Young’s modulus, allowable stress, BMD, maximum force and strain. Between Young’s modulus and all microstructure parameters, especially between BV/TV, bone mineral density (BMD) and Tb.Th, had higher linear regression coefficients R^2^ = 0.5676, 0.6382, 0.3535, respectively. BMD and yield strength, BV/TV, and allowable stress also had better regression coefficients, R^2^ = 0.5227, 0.5259, 0.5426, respectively. Conclusions: The contribution of the microstructure and mechanical properties of the C2–C7 cervical spine to the movement of the cervical spine is different and has a good correlation and the effectiveness of the finite element model is also verified that we can correctly calculate the microstructure and mechanical properties of the cervical articular process to evaluate the stability and injury risk of cervical vertebrae by the established model.

## 1. Introduction

The proliferation and degeneration of the cervical articular process is an important cause of cervical instability and nerve root compression that is regarded as the engine of chronic neck pain [1]. The irregular proliferation and hypertrophy of the articular process can change the morphology of the articular process, which will eventually lead to the change of the structure and stress state of the articular process and the loss of the stability of the cervical vertebrae [2]. Therefore, the study of the microstructure and finite element mechanics of the cervical articular process is of great significance for clinical research and the treatment of related diseases. However, it is rare to report a comparative study on the prediction of apparent mechanics and pathogenicity of the regional microstructure parameters of the articular process of the cervical vertebrae in adults. The anatomical characteristics of the articular process are the anatomical basis of degeneration and traumatic joint disease [3]. In order to adapt to the mechanical environment of the body, there are differences in structure and microstructure between load-bearing bone and no load-bearing bone. Bone quality and structure always exist in the form of an adaptive mechanical load [4]. The density of the overall tibial cortical bone and trabecular bone in athletes shows there is no significant difference between the stress fracture and non-bearing fracture [4], and the microstructure of the anterior, posterior, left and right areas of the tibia are also multivariate according to the bearing force [5]. The volume of dorsal trabecular bone trabecular volume, bone trabecular volume fraction, and bone trabecular connectivity are greater than the ventral side, and the dorsal bone trabecular separation is less than the ventral side [6]. Based on the above literature, the method of finding the microstructure and mechanical changes in different regions of the same site by using the same bone zoning method is reported in our study. The articular process includes cortical bone, cancellous bone, and cartilage, and cancellous bone is also suitable for optimal load transfer by pairing suitable strength and stiffness to minimal weight [7]. Bone mineral density (BMD) remains the most widely used measure of bone quality and fracture risk, but BMD alone cannot fully explain the vertebral strength and rate of fracture incidence [8]. In the past, the microstructure of bone tissue was observed by histology, but now micro-CT is used to evaluate the microstructure of bone in a non-destructive three-dimensional way [9]. In this study, the correlation between the microstructure parameters, bone volume fraction (BV/TV), trabecular number (Tb.N, 1/mm), trabecular thickness (Tb.Th, mm), bone surface/bone volume (BS/BV, 1/mm), trabecular separation (Tb.Sp, mm), trabecular pattern factor (Tb.Pf, 1/mm), and bone mineral density (BMD, mg/cc) of articular process regions and the mechanical parameters of the finite element method were compared and analyzed, and the correlation between the four VOI microstructure parameters and the mechanical parameters of the finite element method discussed. In order to effectively evaluate the risk of degenerative changes and injury caused by excessive stress concentration of the cervical articular process, the present report is as follows (Table 1).

## 2. Materials and Methods

Statement: (i) All test specimens in this study were approved by the Medical Ethics Committee of Inner Mongolia Medical University: Ethical Approval for Biomedical Research: NO.YKD2015049; (ii) Confirmed that all experiments were performed in accordance with relevant guidelines and regulations; (iii) A statement confirming that informed consent to publish identifying information/images was obtained; and (iv) A statement to confirm that all methods were carried out in accordance with relevant guidelines and regulations.

### 2.1. Specimens Prepare

**Figure 1:**
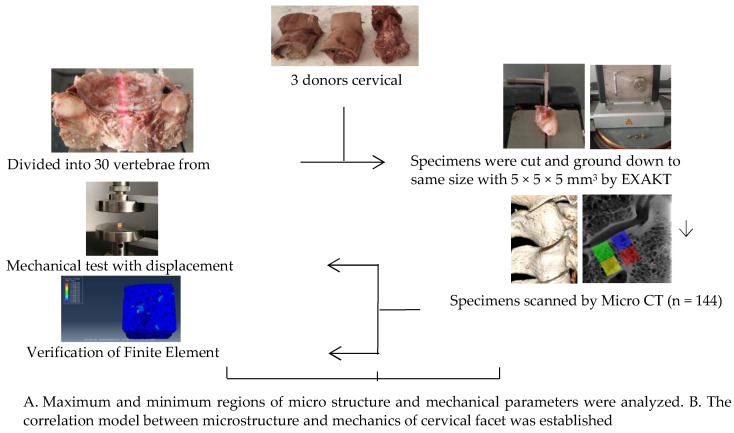
Guide chart of experimental route design.

A total of 144 specimens were selected from three volunteer donors’ cervical vertebrae specimens fixed with formalin (average age 45, provided by the Medical Ethics Committee of Inner Mongolia Medical University: Ethical Approval for Biomedical Research: NO.YKD2015049) were examined by senior doctors without trauma and deformity. The surrounding soft tissue, nerve, and vascular nerve of cervical vertebrae were eliminated, the intact vertebral body and articular process structure were retained of C2–C7, and the intact vertebral body was retained to facilitate the analysis of the angle of physiological curvature of each articular process of C2–C7 (Figure 1). Emphatically, all methods were carried out in accordance with relevant guidelines and regulations.

### 2.2. Micro-CT Scanning

Relying on the micro-CT scanner (Siemens Inveon PET/CT, Germany, Department of Nuclear Medicine, affiliated Hospital of Inner Mongolia Medical University), the prepared complete cervical vertebrae specimens were fixed in the micro-CT high-resolution scanning slot, and the micro-CT scanning warehouse was promoted by the sequence from C2 to C7. Scanning parameters: equipment voltage 80 kV, current 500 mA, spiral scanning mode, x-ray emission source was the variable focal emission source, focus diameter was 6 μm, the maximum resolution was 16.75 μm, and the scanning layer number was 1024 pixels. The image was scanned to “CTA” format storage and post-processed using Inveon Research Workplace 3D reconstruction software (provided by the Department of Nuclear Medicine, affiliated Hospital of Inner Mongolia Medical University, Hohhot, China). Micro-computed tomography (µCT) and scanning electron microscopy (SEM) are vivo characterization techniques that are the gold standard in observing facet joint morphology and degeneration at a microscopic scale [10].

### 2.3. Measurements of Bone Microstructure

**Figure 2:**
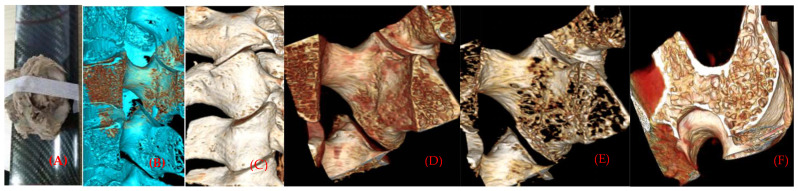
(**A**) Scanning specimens by micro-CT. (**B**,**C**) Three-dimensional reconstruction of the cortical and bone marrow of the articular process. (**D**,**E**) Distribution of articular process trabecular bone with different threshold values. (**F**) Plate-, rod-shaped meshwork bone connection form of articular process cross-section.

The original data of the facet section scanned continuously by micro-CT were imported into the main interface of the Inveon Research Workplace, in a way of no damage and compression. After entering the main interface, general analysis is selected to show the two-dimensional image of coronal, horizontal and sagittal planes, and 512 pixels are selected in all three plane images to observe the microscopic morphology of the specimens and make an analysis and description. Then, go back to the main interface of the Inveon Research Workplace 3D reconstruction software, select MultiModal 3D visualization to display three plane two-dimensional images of coronal, horizontal and sagittal planes, and 3D reconstruction images of specimens (Figure 2A,B).

Selected from Segmentation, Orientation to XZ Plane, we selected four cubes of interest region of each facet bone trabecula and bone marrow with the same threshold binarization with their own software in the horizontal two-dimensional image, with a volume of 5 × 5 × 5 mm^3^. A total of 144 samples on both left and right sides were selected with four interest interval volumes (tissue volume, TV), whose superior articular process was separate to the anterior 1/2 (SP), and inferior 1/2 (SP), and whose inferior articular process was separate to the anterior 1/2 (IA), and inferior 1/2 (IP). However, larger specimens can provide average structural and mechanical properties of a piece of bone, but may not reflect regional structural variations, where failure may occur, which also indicates that the bone structure is region dependent [11].

**Figure 3:**
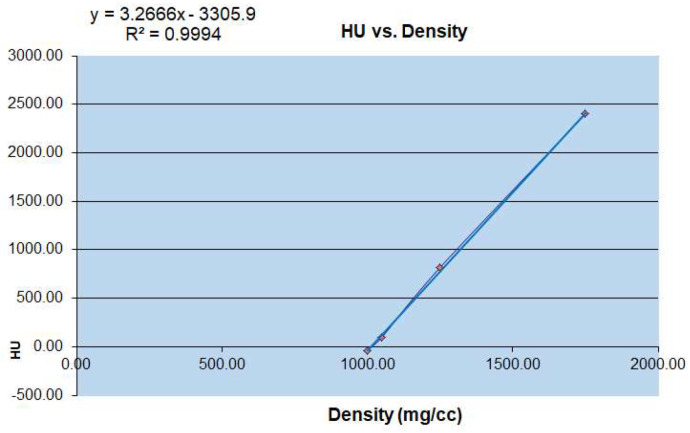
Conversion relationship between micro CT-HU value and bone mineral density (mg/cc).

To obtain the microstructural parameters using micro-CT, the specimens used are often cubes or cylinders, with a volume of about 4–10 mm^3^ [12]. According to the morphology of the physiological curvature of the articular process and the stress state of the articular process, the different vertebral sequences of the articular process are from C2 to C7 left and right lateral (Figure 2). The contribution of microstructure and mechanical properties of the four regions on the right lateral to the physiological activity of the human cervical vertebrae and the evaluation of cervical spondylosis [13]. Micro-computed tomography (micro-CT) has become the gold standard in three dimensional (3D) imaging of trabecular bone structure [14,15]. Moreover, the corresponding bone mineral density was calculated by measured rod voxel values HU with the below formula (Figure 3).

### 2.4. Mechanical Compression Experiment

**Figure 4:**
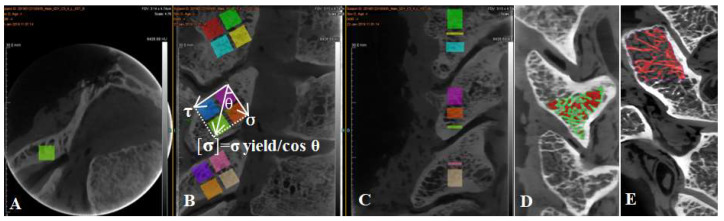
The conversion relationship between allowable stress ([σ]), yield strength (σ yield), shear stress (τ), and regional angle (θ). (**A**) Cross sectional bone trabecular microstructure of articular process. (**B**) Segmented relationship of four regions of articular process. (**C**) Sagittal bone trabecula of articular process. (**D**) The proportion of articular process trabecular bone and bone marrow cavity. (**E**) Threshold segmentation and extraction of articular process trabecular bone.

Each articular process from C2 to C7 was separated from the cervical vertebra, and then the articular process was fixed with a fixture and then cut into four regions, superior anterior 1/2 (SA), superior posterior1/2 (SP), inferior anterior 1/2 (IA), and inferior posterior 1/2 (IP) by a hard tissue slicing machine (Germany, AKATE) (Figure 4). The size of each sample was measured by a micrometer to ensure that the side length and volume of each sample were equal. And ensure the each face was smooth with an emery papering machine with the volume of each sample of 5 × 5 × 5 mm^3^. Because the angle and shape of the articular process of C2–C7 are different, the degree of stress concentration in different areas of the articular process is different (microstructure exists to adapt to the load ability of the articular process because of different microstructures) (Figure 2). The spindle directional stress and shear direction stress are the effects of an alternating load in order to adapt admirably to the articular process at different angles. In general, the anterior and posterior extension of the cervical spine angle is about 45 degrees [16], and the results of the anterior and posterior movement of the upper and lower vertebral bodies are the result of the anterior and posterior movement of the articular process. The upper and lower facet of each articular process is not completely horizontal, which leads to the different direction and magnitude of the force in different facets and even in different areas of the articular process, and leads to different ways and degrees of the articular process being injured [17]. Therefore, in this experiment, each cervical articular process was divided into four regions, and the load generated by the movement of the head and upper cervical vertebrae in each region included the shear stress perpendicular to the joint surface in addition to the vertical downward principal stress. It was found that the resultant force of normal stress and shear stress in each region is the allowable stress in the joint process area. As shown in the following figure, the vertical downward normal stress is made, the shear stress is perpendicular to the articular process region, and the angle between gravity and shear force is θ, so the allowable stress in this region can be calculated following the comparison expression [σ] = σ yield/cos θ (Figure 4).

**Figure 5:**
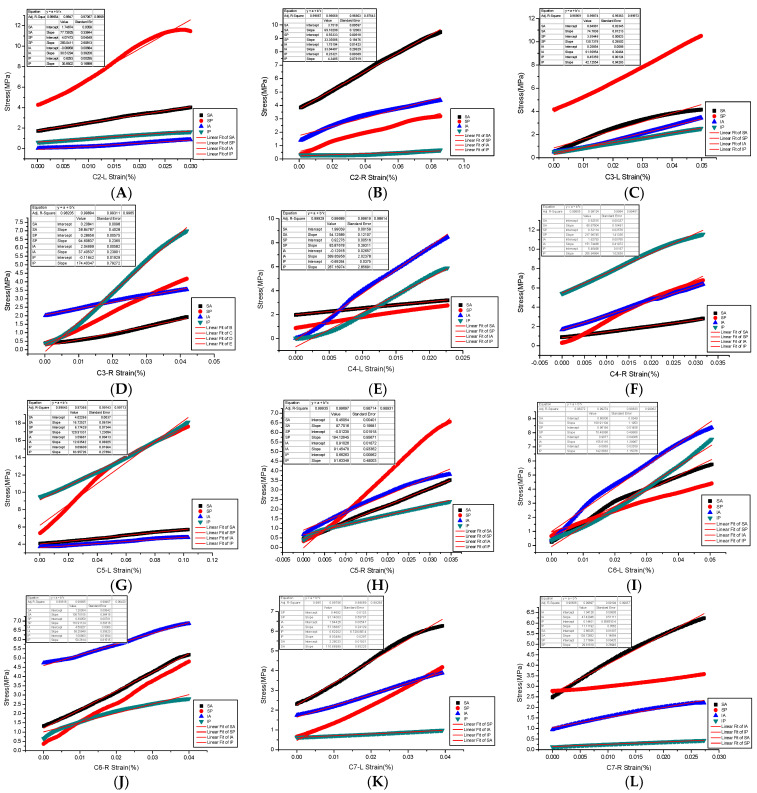
(**A**–**L**) are Young’s modulus fitting of stress and strain in four regions of bilateral articular processes of C2–C7.

The end face of each experimental sample was smoothed to reduce the influence of friction resistance on transverse deformation [18]. The whole process of compression of the sample is controlled by displacement (compression with the strain rate is 8.3 × 10^−9^ m/s). The prescribed displacement is usually exerted as a loading condition in mechanical tests and finite element analysis (FEA) to evaluate the mechanical properties of a trabecular cube [19]. The material compression execution standard is as follows: GB/T 1041-1992 (International implementation Standard for Compression Properties of Plastic Materials). The bone tissue constitutive law was prescribed based on the elastoplastic material model that incorporates geometric large deformations and material non-linearity [20]. The sensitivity and accuracy of force and displacement were detected by the extensometer. The standard distance of the extensometer is the sample diameter (specified non-proportional extensibility). After the mechanical compression test, the maximum applied force, maximum stress, maximum strain, yield strength, and allowable stress of each cervical articular process region sample were obtained. The yield strength is the stress at the lower yield point (stable above the yield point) (Figure 5). Specifically, the yield strength and yield strain were determined using the 0.2% offset technique [21], and the slope was obtained as the elastic modulus of the specimen in this area by fitting the stress-strain curve [20]. We found that most of the samples were compressed into a diamond shape after mechanical testing.

### 2.5. FEA Simulation

The original DICOM format data scanned by micro-CT were imported into Mimics software (16.0) without loss and damage. We applied the same predefined thresholds set to separate the articular process bone trabeculae, bone cortex, and bone marrow. Then, the bone trabeculae and bone cortex of four regions of interest were extracted from the target articular process by regional growth technique and calculate three-dimensional images from a mask with a high quality of four regions of the complete articular process. The 3D object images for each region were redrawn through a 3-Matic grid including the following steps. The first step is to mesh, and auto remesh (parameters, shape quality was 0.5, maximum geometry was 0.5, maximum triangle was 0.1); quality preserving reduces triangles. The second step is used to fix wizards to diagnose the whole model in order to ensure all bad edges and noise shells have been removed. Third, create a volume mesh (parameters, the maximum edge was 0.1) that is copied to the Mimics and select the FEA mesh for which you want to assign materials. Each FEA mesh of the articular process region contains 133,742 elements. The properties of the FEA model material are assigned by the following formula [21,22].

Density = 0.00097 × GV(1)

E-Modulus = 19.04 × Density^1.64^ (2)

Poisson ratio = 0.3(3)

Next, export the FEA models to the ABAQUS with the inp. files output format, then the FEA models are introduced into ABAQUS to edit the material behavior. The density distribution is the same and the material properties for all FEA models were assumed to be linear and isotropic, and the plastic hardening attribute is isotropic. Moreover, the finite element loading of the articular process in the FEA models was calculated with the following steps. First of all, assemble the model module; create the analysis step and field output; and finally, the static program type. The output variable selects the stress, strain, displacement, force and reaction force, and the output process variable selects the energy. The FEA models of the articular process are created to the load surface that is the upper surface of the articular process model, and each node loads uniformly one by one; the whole process of compression of the model is controlled by displacement (compression with the strain rate is 8.3 × 10^−9^ m/s), similar to the mechanical test in the *Z*-axis. The last step is to create boundary conditions in the bottom with completely fixed and submit jobs in Job Manager after creating a job, which was our FEA model. However, this material model can be used to predict the apparent level yield behavior of trabecular bone [23].

### 2.6. Statistical Analysis

IBM SPSS 16.0 software was used to analyze the data, and the Bartlett test was used to test the homogeneity of variance. Univariate multivariate ANOVA was used to analyze the influence of three groups of independent variables on the microstructural parameters of the articular process of dependent variables of C2–C7 with different vertebral order, right and left lateral articular process, and four regions of each articular process. It can not only analyze the effect of a single factor of the independent variable (main effect), but can also analyze the interaction between factors (interaction effect). If the main effect is significant (the difference between groups), there is a significant difference between two or more levels of factors. After the event, we can continue to compare the mean difference between multiple levels of the same factor, which is called multiple comparison or simple effect test by fixing one of the independent variables at a certain level and examining the influence of the other independent variable on the dependent variable in SPSS (IBM SPSS Statistics 19) with a “MANOVA” command. If the significance is less than the significance level (the general significant level was set to 0.05), it is considered that there are significant differences in the overall mean value of the control variables at different levels. Furthermore, multivariate regression analysis was performed to elucidate the microstructural parameters of the cervical articular process most strongly related to estimated bone strength by ridge regression analysis.

## 3. Results

### 3.1. Regional Variation Microstructure of Articular Process

If the main effect is significant (inter-group difference), it indicates that there is a significant difference between the two or more levels of the factor. The intersubjectivity effects of the four regions in the articular process were analyzed and showed significant differences in BV/TV, BS/BV, Tb.Sp, and Eta of 5.5%, 4.8%, and 4.3%, in which different regions of the independent variable articular process had the greatest influence on BV/TV value (Eta% as a percentage of each factor for TV/BV, indicate the level of value). There were significant differences in BV/TV, Tb.N, BS/BV, Tb.Th, Tb.Sp, and Tb.Pf among the main effects of articular process in different orders of C2–C7. The partial Eta of Tb.N was the largest, which indicated that the joint process of different vertebral order had the greatest influence on the value of Tb.N. There is only a significant difference in Tb.Th between the left and right laterals. The partial Eta was 2.0%, which indicated that the difference between the left and right groups had the greatest influence on Tb.Th.

Simple effect analysis, The interaction effect between different regions of the articular process and different vertebral levels was only significantly different from the Tb.N results. The interaction effect was 14.4% that has the greatest influence on the Tb.N value. There was no significant difference in the microstructure parameters between intra-articular regions and lateral interaction. The effect score of interaction effect on the BV/TV results was 2.4%. There was no significant difference between the left and right side and different vertebral order. The effect score of interaction effect on Tb.Pf was 2.6%. There was no statistically significant difference in the interaction effects between the three independent variables in different regions and in the left and right sides. The maximum influence score of the interaction effect on the BS/BV result value was 4.9% (Table 2).

**Figure 6:**
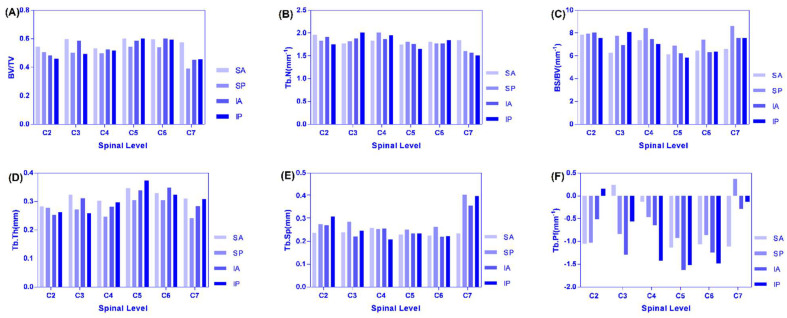
(**A**–**F**) are distribution and variation trend diagram of each microstructure parameter in four regions of C2–C7.

Analysis of the changing trend and regularity of six microstructure indexes in the four regions of the left and right lateral of C2–C7 that increased from C2–C4 to C5–C6 and decreased C6–C7, and C7 was the smallest for BV/TV (Figure 6A). Because the cervical vertebrae supply blood vessels, the vertebral artery goes upward through the C6 to C1 transverse foramen of the cervical vertebrae, except that the transverse foramen of the cervical vertebrae is small in the C7 cervical vertebra and all the other vertebral arteries pass through the transverse foramen of the cervical vertebrae. In particular, the intervertebral foramen and transverse holes of the seventh cervical vertebrae are the smallest, the nutrient vessels are fine, and the blood supply of the articular processes is relatively small, resulting in a small BV/TV value of the articular processes [24]. The BV/TV value of the cervical vertebrae from the bilateral articular process of C2–C7 was different, and the BV/TV value of articular process from the left lateral of C2–C7 was larger than that of the right lateral. From the point of view of anatomical structure and hemodynamics, it is revealed that the left common carotid artery and subclavian artery directly originate from the arch of autonomic artery and receiving higher pressure during the occurrence of vertebral artery diameter, whose blood flow on the left side is significantly larger than on the right side, so the left vertebrae and appendages including the joint process have a richer supply of blood flow; in addition, the angle between the left and the subclavian artery is smaller than that on the right, and blood supply of thee vertebral artery is dominant on the left lateral [24].

The maximum region on the left and right lateral were all SA regions. From C2 to C4, the IP region of the left was the smallest and the SP region of right was the smallest. From C2 to C4, the IP region of the left was the smallest and the SP region of right was the smallest. It can be seen that from C2 to C7, BV/TV was small in the posterior region of the articular process (the activity of the cervical vertebrae is mainly in normal physiological curvature or protruding state, and the accumulated load in the anterior area of the joint process was larger than that in the posterior regions). The maximum value of TB.N was mostly concentrated in the SA and SP regions, and the minimum value was concentrated in the IP region, and the changing trend was the same as that of the decreasing trend of C2–C5, the increase of C5–C6, and then obvious decrease of C6–C7 (Figure 6B). There were similar trends of SA, SP, and IA regions for BS/BV where C2–C3 decreased and C3–C7 increased. However, C3–C5 decreased and C5–C7 increased in the IP region. We found that the overall variation tendency was opposite to the BV/TV, the maximum value was mostly concentrated in the SP region, and the minimum value was mostly concentrated in the SA region. The reason is that when SB is constant, the larger the BV and the smaller the SB/BV ratio, which accords with the variation law between the microstructure parameters of this experiment (Figure 6C). The left side of Tb.Th where C2 was larger than the right lateral, and the variation of SP > SA > IA > IP, was similar to BV/TV (Figure 6D). The maximum region was concentrated in the SA region. The values of the C5 articular process reached the peak value, and the variation tendency was similar between the left and right lateral. The maximum and minimum values of Tb.Sp were mostly concentrated in the SP and SA regions (it is suggested that the bone structure is suitable for function and has obvious anisotropy) (Figure 6E). From C2–C3 increased and to C3–C7 decreased in SA, SP, and from C2–C4 to C4–C7, it decreased at first and then increased in the IA and IP regions for Tb.Pf. Most of the maximum and minimum values were concentrated in the C7 and C5 levels (Figure 6F).

### 3.2. Simulation and Calculation of Finite Element Models in Articular Process Regions

**Figure 7:**
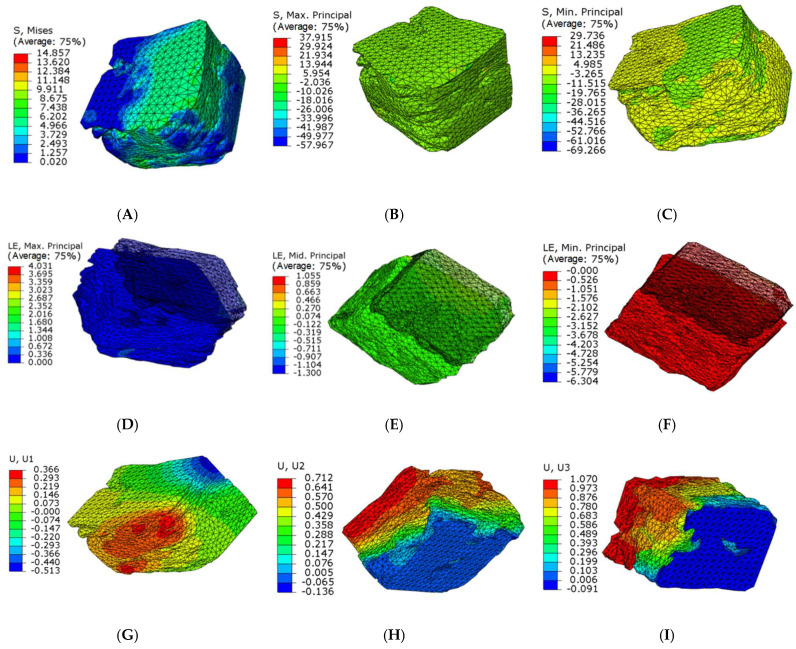
Microfinite element stress-strain nephogram in the SA articular process regions at level C4 right lateral. (**A**) 75% Mises mean stress; (**B**) Maximum stress nephogram; (**C**) Minimum stress nephogram; (**D**) Principal plane maximum real strain; (**E**) Principal plane middle real strain; (**F**) Principal plane minimum real strain; (**G**) X axial displacement nephogram; (**H**) Y axial displacement nephogram; (**I**) Z axial displacement nephogram.

**Figure 8:**
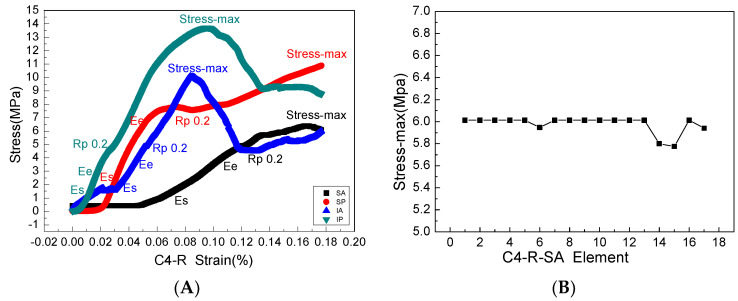
(**A**) is a complete mechanical elastic and plastic stress-strain process in the four areas of the right articular process of the fourth cervical vertebra, where Es, Ee, Rp0.2, stress-max is the elastic starting point, elastic termination point, yield point, and maximum stress point, respectively, and the slope of each stress-strain curve is Young’s modulus. (**B**) The maximum stress calculated by the finite element model is consistent with the mechanical experiment.

The stress and strain values of four regions of each articular process after the mechanical compression experiment were input into the ABAQUS program of finite element simulation, where emphatically, the results of stress and strain output after submission were consistent with the experimental results of mechanics, which fully verifies the effectiveness of the finite element models. Through the analysis of the output results of the finite element, the range value of 75% Mises stress in the SA region at level C4 of the right lateral articular process was 1.257–7.438 MPa (Figure 7A) and the mean value was 6.01375 MPa (Figure 8B); this value is similar to the results of the mechanical compression experiments (Figure 8A), and the effectiveness of the model was verified.

Because the articular surface of the cervical joint is at an angle of 45 degrees with the horizontal position and the angle of the joint regional is also different, which results in complex forces including principal stress and shear stress. In addition, transverse and longitudinal residual stresses of the articular process are gradually transformed from compressive stress to tensile stress; furthermore, the longitudinal residual stress is in the region away from the main force center of the joint process, then the transition from tensile stress to compressive stress is close to the yield strength of the articular process. The change regulation of residual stress in different layers is different, while that for transverse residual stress is gradually transformed from compressive stress to tensile stress. For the longitudinal residual stress, it also gradually transitioned from compressive stress to tensile stress in the region near the principal stress, however, with the region far from the principal stress, the tensile stress gradually transitioned to the compressive stress. Accordingly, ranges of the maximum and minimum principal tension at level C4 right lateral in SA region were −26.066~13.944 Mpa and −19.765~4.985 Mpa, (Figure 7B,C). The range of the principal plane maximum real strain, the middle, and the minimum real strain were 0%~67.2%, −10.026%~13.944%, −11.515%~4.985%, respectively (Figure 7D–F). The displacement ranges along the direction of X, Y, and Z axis were as follows: −0.513~0.366 mm, −0.136~0.712 mm, −0.091~1.070 mm, respectively(Figure 7G–I).

### 3.3. Establishment of Correlation between Micro-CT Microstructure and Mechanical Properties Parameters of Mechanical Compression Test

**Figure 9:**
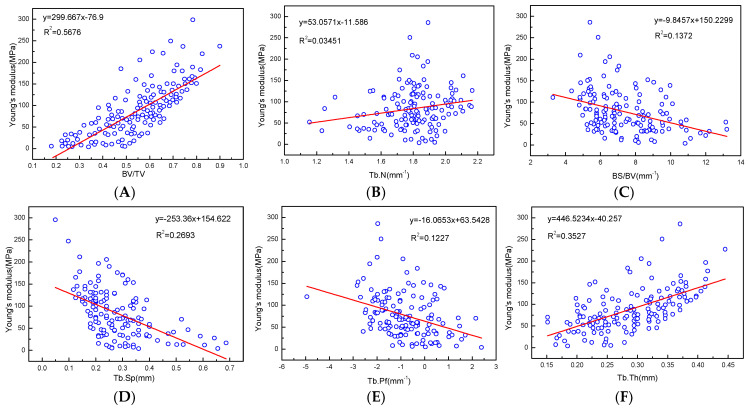
(**A**–**F**) are linear correlation between microstructure parameters and Young’s modulus fitting.

Young’s modulus has a good positive correlation with all microstructure parameters, and the regression coefficients with BV/TV, BMD, and Tb.Th were relatively high at R^2^ = 0.5676, 0.6382, and 0.3535, respectively (Figure 9, Table 3).

There were negative correlations with Young’s modulus and BS/BV, Tb.Sp, and Tb.Pf, even though the correlation was low. There were also correlations between the mechanical properties parameters of the articular process, which had better positive linear correlations between BMD and yield strength, BV/TV, and allowable stress, R^2^ = 0.5227, 0.5259, and 0.5426, respectively. There were negative correlations between force (max) and Tb.Sp, BS/BV, Tb.Pf, and stress (max), BV/TV and strain (max), although the correlation was relatively low (Table 3).

## 4. Discussion

Cervical articular processes, which are requisite structures to maintain cervical stability and transfer load, have a complex structural shape and stress state; moreover, abnormalities also lead to serious clinical symptoms. In 1983, Denis et al. put forward the three-column theory of spine that the posterior column bears 20% of the cervical motion load, and the articular process belongs to the main stress structure of the posterior column, which lays a biomechanical foundation for the treatment of lower cervical spine injury [25,26]. This is the first study of the correlation between the regional microstructure of the cervical articular process and the mechanical properties of finite element in adults to guide the safety protection of cervical physiological activities, diagnosis, treatment, and the exploration of bone joint bionic tissue engineering of the articular process provide a valuable reference. The results of this study show that the cervical articular process is the main bearing site of cervical vertebrae, second only to the vertebral body, which loads the contribution of four regions, different vertebral sequences, and the left and right articular processes to the whole cervical physiological activity are not the same. The maximum and minimum value of the C2–C7 articular processes and regions were C5 and C7, SA, and P regions for BV/TV, TB.N, and Tb.Th, which had the same variation tendency for four regions and two laterals although the left lateral value was greater than the right. The anterior flexion and extension of the cervical spine were centered from C4–5 to C5–6, where the stress was concentrated and large. Consequently, proliferation was earlier and more common, and C5 was the most obvious [27]. Studies are also supported by the fact that the region of the spinal cord most affected by CSM (cervical spondylotic myelopathy) (levels of C5–C7) is also the area with the most vulnerable vascular supply by affecting blood flow on bone growth [28]. Furthermore, the left common carotid artery, subclavian artery directly from autonomic artery arch, the diameter of vertebral artery, and the blood flow on the left lateral were significantly larger than those on the right lateral due to higher pressure during occurrence, therefore, the blood flow of the left vertebrae and appendages including the articular process was more abundant, and the angle between the left vessel and the subclavian artery was smaller than that on the right (anatomical characteristics and hemodynamic principles of cervical vessels) [29]. There was a correlation between elastic modulus and all microstructure. There was a positive linear relationship with BV/TV, BMD, and Tb.Th; meanwhile, the trabecular rod length was significantly correlated to the traditional parameter, Tb.Sp, which is a negative determinant of Young’s modulus of human trabecular bone [30]. We also found BMD and yield strength, BV/TV, and allowable stress were relatively good, R^2^ = 0.5227, 0.5259, and 0.5426, respectively. The plate bone volume fraction (pBV/TV) and axial bone volume fraction (aBV/TV) calculated correlated the best with elastic modulus (R^2^ = 0.96~0.97) and yield strength (R^2^ = 0.95~0.96) [31]. A study suggests that with increasing bone quantity, surface area, Tb.N, and Tb.Sp, the amount of force required to fracture the specimen increases, while there was a negative correlation between critical stress intensity values and BS/BV, which suggests that as the specific surface area increases, the critical stress intensity decreases [32]. Similarly, Wegrzyn et al. performed a compression breaking test of the lumbar spine and reported that the BV/TV was strongly correlated with vertebral strength (Pearson’s correlation coefficient, R^2^ = 0.69), but the correlation with Tb.Th was low (R^2^ = 0.24) [33]. The connectivity of bone trabeculae is also important to control the mechanical properties of bone cereals. Based on the morphological parameters of bone trabeculae such as BV/TV of rod and plate bone, they are highly correlated with bone load capacity, Young’s modulus, and yield strength [34]. Furthermore, it is important to confirm the maximum and minimum values of the mechanical properties parameters from four regions of each articular process of C2–C7 to evaluate the maximum force area (stress concentration area) and the largest area of joint process force for the prediction of fracture, degeneration of joint process, and risk of cervical spondylosis caused by changes in cervical articular process microstructure and mechanical properties. We found that the maximum and minimum regions of Young’s modulus, stress (max), and BMD were consistent with TV/BV in both SA and P regions (except for C5 in IA and C7 in IA of Young’s modulus and BMD). Moreover, the maximum and minimum value of allowable stress were mostly in the SP and IP regions (except for C7 in the IA region) The results show that the articular process had a large allowable stress in the SP region so that the risk of injury is small, however, the allowable stress in the IP region is small and more subjected to shear stress. Meanwhile, the risk of damage is relatively high. Regional minimum BV/TV will predict the maximum ultimate stress ability of bone to assess the risk of fracture due to excessive stress concentration at the facet segments of the cervical spine [35]. In this study, the changes of microstructure and finite element model of different facets, vertebrae sequence, and regions of articular processes in adults were observed that contribute to the analysis of stress and stress threshold to assess the risk of cervical articular process injury. Moreover, according to Wolff’s law, bone mass and structure always exist in the form of adaptive mechanical load that in mechanical tests and finite element analysis, the trabecular structure is usually evaluated by loading conditions such as stress and strain [36]. However, the bone trabecular model can be used to predict the apparent yield behavior of trabecular bone [37]. In our study, the yield stress accounted for 58.95% of the maximum stress, and the yield strain accounted for 39.49% of the maximum strain. The bilinear tissue constitutive model was used to describe the nonlinear properties of bone tissue materials. The tissue compression strain and tensile yield strain were 0.8% and 0.48%, respectively. The majority brittle fracture is directly caused by osteoporosis and usually occurs in the dominant part of bone trabeculae (cancellous bone).

The main limitation of the presented methodology is that specimens were fixed with formalin and may differ from fresh specimens, however, there are few studies comparing the effects of different preservation time on the microstructure and mechanical properties of bone in different parts of the body, which is also an important issue that we will explore in later experiments. Another limitation is that the microstructure parameters of this study were relatively few (because of the limitations of the micro-CT device), meanwhile, in the next study, we will add more microstructures such as Euler number, connectivity, connectivity density, degree of anisotropy, and structure model index, which will be a more comprehensive evaluation of the relationship between the parametric parameters of the trabecular bone and the mechanical structure.

## 5. Conclusions

In conclusion, the structural morphology and stress state of the cervical articular process is complex, and it is also an important structure to maintain the stability and transfer the load of the cervical vertebrae. Significantly, the abnormality of the articular process leads to various serious clinical symptoms. This study not only accurately locates the microstructure and the load performance, moreover, the maximum and minimum regions of the C2–C7 articular process. Furthermore, it effectively evaluated the risk of injury and cervical spondylosis in each cervical articular process due to differences in microstructure and mechanical properties of different regions.

## Figures and Tables

**Table 1:** Significance of micro-CT microstructure parameters and its relationship with clinical application.

Microstructure Parameters	Meaning	Relationship
Trabecular volume fraction, BV/TV	The ratio of bone trabecular volume to an area of interest volume	Positive correlation with bone load capacity and negatively correlated with osteoporosis, fracture, hyperplasia, and degeneration [6]
Trabecular Number, Tb.N, mm^−1^	Number of points of intersection between bone tissue and non-bone tissue	
Trabecular Thickness, Tb.Th, mm	The average thickness of bone trabeculae	
Bone Surface/Bone Volume, BS/BV, mm^−1^	The ratio of the surface area of bone trabeculae to the volume of bone cerebellum	Negatively correlated with bone quality, when osteoporosis occurs, the value increases
Trabecular Spacing, Tb.Sp, mm	The average width of the pulp cavity between the trabecular medulla	
Trabecular Pattern Factor Tb.Pf, mm^−1^	Representing bone trabeculectomy connectivity	

**Table 2:** Multiple comparisons of microstructure parameters between four areas of the articular process left and right, and intervertebral sequence (univariate analysis of variance-intersubjectivity effect test, F, Eta%, *n* = 144).

			Regions		Lateral		Spine Level
		Lateral		Lateral		Spine Level	
	Spinal Level						
BV/TV	5.01 *, 13.8	1.59, 2.4	3.74 *, 5.5	5.00, 4.8	3.78 *, 1.9	0.28, 0.7	5.00 *, 11.5
Tb.N	3.27 *, 6.1	0.54, 0.8	2.39 *, 4.6	2.15 *, 14.4	0.52, 0.3	0.47, 1.2	9.43 *, 19.7
BS/BV	6.15 *, 12.9	0.77, 1.2	3.22 *, 4.8	0.52, 3.9	3.18, 1.6	0.32, 0.8	3.98 *, 9.4
Tb.Th	4.22 *, 13.2	0.81, 1.2	2.42 *, 4.5	0.58, 4.4	4.01 *, 2.0	0.14, 0.4	4.69 *, 10.9
Tb.Sp	4.91 *, 12.7	1.06, 1.6	2.85 *, 4.3	1.25, 8.9	1.86, 1.0	0.40, 1.0	7.73 *, 16.8
Tb.Pf	4.25 *, 13.1	0.93, 1.4	1.01, 1.6	1.58, 11	1.12, 0.6	1.04, 2.6	3.88 *, 9.2

* The test of bivariate effect is statistically significant, *p* < 0.05. Eta% is the percentage of variables that interact with each other, and the greater the value, the greater the impact.

**Table 3:** Linear regression relationship between the microstructure and mechanical properties.

Parameters	*p*-Value	Adjusted R-Squared	Linear Regression Equation
BV/TV & Young’s modulus	0.000 ^a^	0.5676	y = 299.667x − 76.9
Tb.N & Young’s modulus	0.015 ^a^	0.0354	y = 53.0571x − 11.586
BS/BV & Young’s modulus	0.000 ^a^	0.1372	y = −9.8457x + 150.229
Tb.Sp & Young’s modulus	0.000 ^a^	0.2693	y = −253.36x + 154.622
Tb.Pf & Young’s modulus	0.000 ^a^	0.1237	y = −16.0653x + 63.5428
Tb.Th & Young’s modulus	0.000 ^a^	0.3535	y = 446.5234x − 40.257
Density & Young’s modulus	0.000 ^a^	0.6382	y = 202.9904x − 191.3834
BV/TV & Force(max)	0.000 ^a^	0.3260	y = 1194.207x − 209.975
BS/BV & Force(max)	0.000 ^a^	0.2702	y = −1493.28x + 815.159
Tb.Sp & Force(max)	0.008 ^a^	0.0411	y = −710.25x + 516.253
Tb.Pf & Force(max)	0.031 ^a^	0.0263	y = −45.527x + 287.201
Density & Force(max)	0.015 ^a^	0.0341	y = −16.0653x + 63.5428
Tb.Pf & Stress(max)	0.015 ^a^	0.1035	y = −1.905x + 7.227
Tb.Th & Stress(max)	0.000 ^a^	0.1672	y = 10.205x − 7.925
Density & Stress(max)	0.000 ^a^	0.4531	y = 0.014x + 0.478
BV/TV & Strain(max)	0.026 ^a^	0.0844	y = −361.195x + 2.48
Density & Strain(max)	0.099	0.0124	-
Density & Yield Strength	0.000 ^a^	0.5227	y = 11.9x − 11.799
BV/TV & Density	0.000 ^a^	0.5259	y = 1.006x + 0.935
Tb.N & Density	0.000 ^a^	0.3932	y = 0.634x + 0.22
Tb.N & Stress(safe)	0.084	0.0437	-
Density & Stress(safe)	0.000 ^a^	0.5426	y = 18.962x − 19.253

*p*-value ^a^ Pearson’s correlation coefficient: *p* < 0.05.

## Data Availability

Data sharing is not applicable to this article.
